# A cost efficient protocol to introduce epitope tags by CRISPR-Cas9 mediated gene knock-in with asymmetric semi-double stranded template

**DOI:** 10.1016/j.mex.2021.101365

**Published:** 2021-04-27

**Authors:** Qi Fang, Yoichi Shinkai

**Affiliations:** a Cellular Memory Laboratory, RIKEN Cluster for Pioneering Research, Wako 351-0198, Japan; b Graduate School of Science and Engineering, Saitama University, Saitama, Japan

**Keywords:** Homology directed repair (HDR), Gene editing, Epitope tagging, Semi-doublestranded oligodexynucleaotide (semi-dsODN)

## Abstract

To study the biological function of uncharacterized proteins, specific antibodies are in high demand. However, the production of desirable antibodies such as highly specific or high affinity is not always successful. Furthermore, even if commercially available antibodies exist, the cost, quality, and accessibility often differ from country to country. In comparison, epitope tags are reliable and economical options since good antibodies against major epitope tags are commercially available. Although exogenously expressed epitope-tagged protein appears as a timely method, the excessive protein production may not faithfully recapitulate its biology. Thanks to the recent advances in genome editing by CRISPR-Cas9, HDR-mediated endogenous protein tagging has become an accessible approach for many labs. However, currently the synthesis of long (>100 bp), chemically modified oligos can be time-consuming and costly. To develop a reliable, simple, and cost-effective epitope-tagging method that requires minimal materials and apparatus, we focus on an approach utilizing two non-chemically modified shorter-annealed oligos (semi-dsODNs) mediated HDR for epitope tags insertion. We also use a cationic lipid chemical, polyethyleneimine (PEI), for plasmid delivery to minimize the cost and materials used while a considerable success rate could be achieved.

• This protocol provides a more economical way to generate CRISPR-Cas9 mediated gene knock-in.

• This protocol provides a simplified design of semi-dsODN without chemical modification on the oligos.

• This protocol provides a simplified experimental procedure. In vitro assembled Cas9 complex and electroporation are not required.

## Graphical abstract

**Figure:**
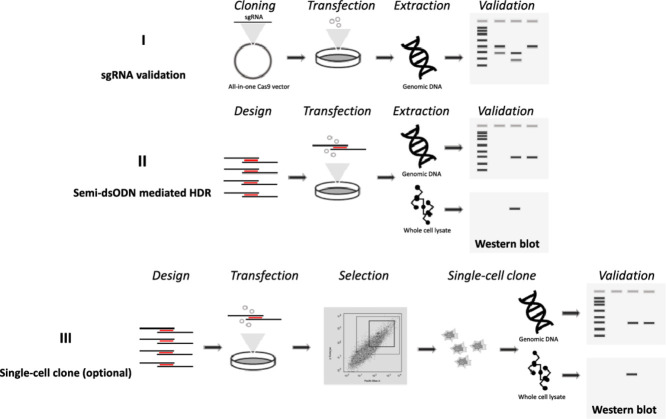

Specifications Table

**Table:** 

Subject Area:	Biochemistry, Genetics and Molecular Biology
More specific subject area:	CRISPR-Cas9 mediated gene editing
Protocol name:	CRISPR-Cas9 mediated gene tagging with asymmetric semi-double stranded template
Reagents/tools:	1.1 Materials (can be purchased from any supplier unless specified in the protocol) MilliQ water/ddH2O sgRNA expression Cas9 vector Restriction enzyme Gel extraction kit sgRNA oligos DNA ligation mix Chemical competent Mach1/DH5alpha LB with ampicillin Plasmid prep kit Primers Nanodrop spectrophotometer Agarose (powder) TAE buffer DNA ladder DNA dye HeLa cells (or others) DMEM (High Glucose) FBS L-glutamine Pen-Strep PEI Opti-MEM Trypsin PBS Mutation Detection Kit SDS lysis buffer Protein ladder Laemmli buffer Tris/Glycine buffer with 0.1% SDS Tris/Glycine buffer without SDS Tris-Glycine gel (8%) Methanol PVDF membrane Filter paper Skim milk TBS-T Antibodies ECL solution X-ray film FLAG-M2 agarose gel TOPO TA Cloning Kit for Sequencing 1.2 Tools ApE plasmid Editor DNA electrophoresis chamber Water Bath Shaker Heat Block Thermocycler (PCR machine) Refrigerated centrifuge Ultrasonic disruptor Protein gel electrophoresis chamber Western blot transfer chamber Film cassette X-ray film imager 1.3 Optional tools Flow Cytometer (BD FACS Aria) Confocal microscope system (Olympus FV3000)
Experimental design:	HeLa cells were co-transfected with sgRNA expressing Cas9 plasmid and semi-dsODN template by PEI. Genomic DNA was then extracted to validate insertion of epitope tag sequence using PCR. After observing the designated PCR product, the whole-cell lysate was used to validate epitope-tagged protein by immunoprecipitation and Western blot.
Trial registration:	N/A
Ethics:	N/A
Value of the Protocol:	• Low-cost. This protocol simplified the design of semi-dsODN, so no modification on the oligos is required. • Accessible. This protocol simplified the experimental procedures. In vitro assembled Cas9 complex is not required. A commonly used PEI transfection is sufficient.

## Description of protocol

**Fig. 1:**
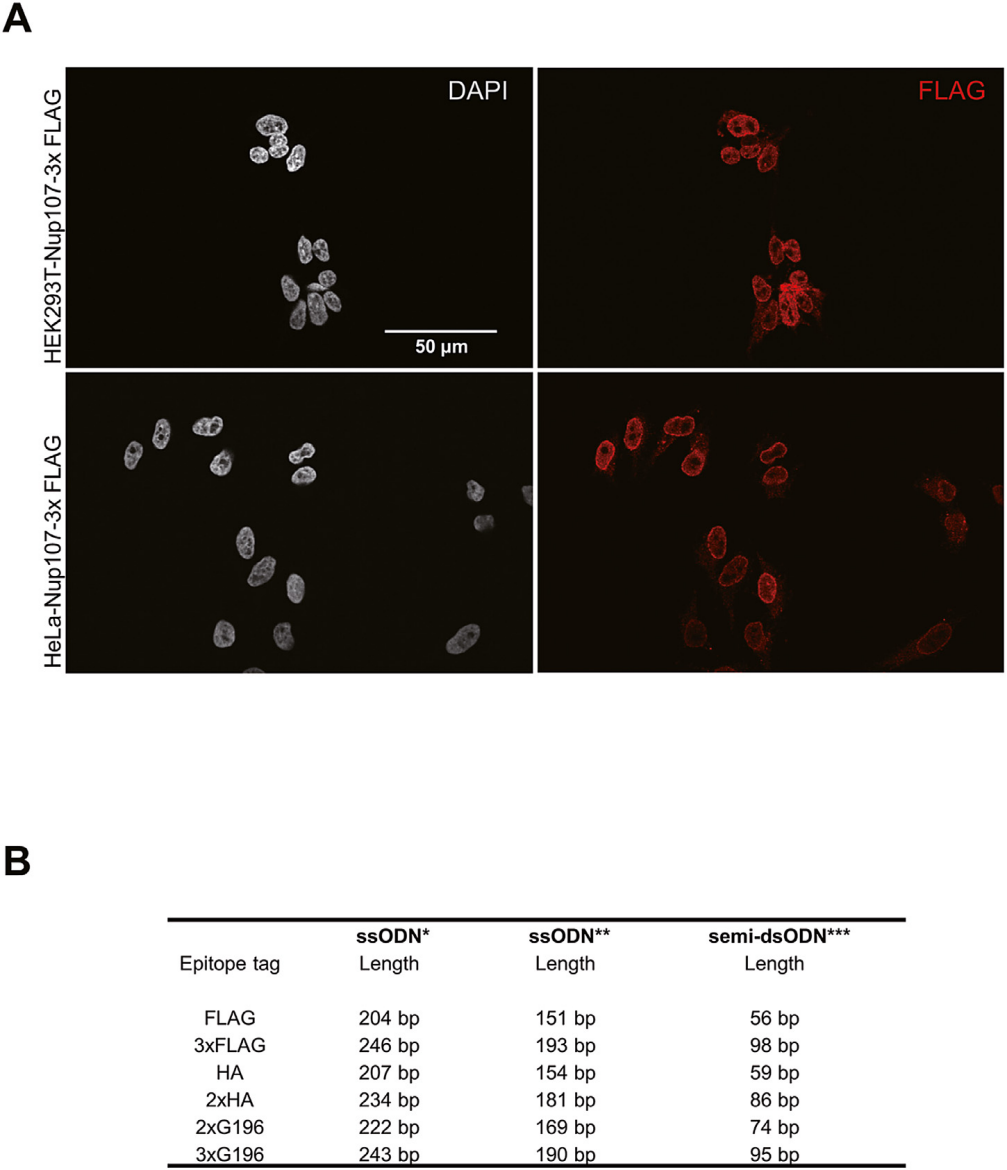
Immunofluorescene imaging of exogenously expressed proteins and comparison of HDR templates from previous publications A) Nup 107-3*FLAG overexpression in HEK293T and HeLa cells. Constructs expressed Nup 107 proteins were delivered by PEI. B) HDR templates of common epitope tags. Oligo lengths shown here were calculated according to previous publications;* a 90-bp homology arm on both sides [3]; ^⁎⁎^36 bp on the PAM-distel side and 91 bp on the PAM-proximal side [2]; ^⁎⁎⁎^a 32-bp homology arm on one side [1].

Epitope tags are potent approaches for studying specific protein functions where there is no reliable antibody available. Although it is possible to express the epitope-tagged protein in mammalian cells exogenously, the biology of overexpressed protein is often incompetent due to its expression level (Fig. 1A). Nowadays, the widely used CRISPR-Cas9-mediated HDR plays a vital role in genome editing, and endogenous protein tagging has become more efficient and approachable. However, HDR template containing epitope tags and homology arms often exceeds 100 bp in length, making this approach costly (Fig. 1B). In 2017, Liang et al. further improved this method using a short double-stranded DNA oligonucleotide with 3′ overhangs [1]. This approach dramatically reduces the length of HDR templates while a significant success rate is maintained. However, this protocol requires electroporation and in vitro assembled Cas9 complex, which again adds cost.

Our modified protocol provides a more affordable, accessible, and simple approach with only minimal performance is sacrificed. In brief, we established a protocol using non-chemically modified semi-dsODNs with common cationic lipid-mediated transfection to achieve endogenous protein tagging.

Step I: sgRNA design, cloning, and validation (cloning two days, transfection and validation three days).

1.1 Design and order sgRNA oligos from the provider of your choice (preferably in 100 µM TE solution). Preferably, the DNA double-strand break (DSB) mediated by Cas9 (3–4 nt upstream of the PAM) should be within a 10-bp range of your insertion site.

1.2 Prepare of sgRNA expression Cas9 vector (pL-CRISPR.EFS.tRFP, Addgene #57819) using BsmBI-v2 (NEB #R0739) digestion following the manufacturer's protocol (please see note). After electrophoresis (0.8% agarose), purify the digested vector using a gel extraction kit. The digested vector can be store at −20 °C for at least a year.

*Note: There are BbsI sites in the tRFP sequence. Please ensure a proper enzyme is used. Also, please extend the digestion time to 2* *h if BsmBI (NEB #R0580) is used. For elution, every 1* *µg of a plasmid is used for digestion can be eluted with a final elution volume of 50* *µl. Also, the uncut plasmid should be run alongside the linearized plasmid. Successfully linearized plasmid appears higher molecular weight comparing to the uncut.*

1.3 Mix 10 µl (100 µM) of both strands of sgRNA oligos (total volume 20 µl) and anneal oligos using settings below:

95 °C for 5 min; ramp down to 85 °C at 0.1 °C/s, hold for 30 s; ramp down to 25 °C at 0.1 °C/s.

Dilute 5 µl of annealed oligos into 195 µl ddH2O (total volume 200 µl). This diluted sgRNA duplex is used for cloning the sgRNA expressing Cas9 construct.

1.4 Mix 0.5 µl of the linearized vector with 2 µl diluted oligos. Set up a ligation reaction using Ligation Mix (TaKaRa #6023) following the manufacturer's protocol.

1.5 Add 20 µl chemically competent Mach1 or DH5alpha directly into the ligation mixture. Mix and incubate on ice for 5 min, heat-shock at 42 °C for 1 min, then return it on the ice for 1 min. Plate the mixture onto an Amp+ LB plate (100 µg/ml) and incubate overnight at 37 °C. A negative control should be set up using the empty linearized vector.

*Note: It is normal to see few colonies appeared the next day on the negative control. However, a successfully made construct should have many more colonies.*

1.6 Pick a few colonies (2–4) and prepare mini-prep for Sanger sequencing using the human U6 – fwd primer

*Note: if another plasmid is used, please choose a different primer accordingly.*

1.7 Seed 0.5 x 10^6^ HeLa cells (ATCC CCL-2) onto a 6 cm dish in DMEM medium one day before transfection. For each dish, transfect cells using the following sgRNA Cas9 construct 2 µg

PEI (Polysciences #24765-2) 8 µl (1 mg/ml)

Opti-MEM (Thermo Fisher Scientific #31985070) 100 µl

Mix and Incubate at room temperature for 5 min

Add this mixture evenly onto the dish and change medium the next day.

*Note: If different cell lines are used, please optimize cell confluence at ~70% on the day of transfection. Also, if the cell line used is sensitive to PEI, other suitable transfection reagents should apply. We routinely use PEI for HeLa and HEK293T (ATCC CRL-1573).*

1.8 At 48 h post-transfection, harvest transfected cells by trypsin and extract crude genomic DNA using the Guide-it Mutation Detection Kit (Clontech #631448) with a slight modification to the manufacturer's protocol. For 1–2 × 10^5^ cells, we usually use 36 µl Buffer I and 4 µl Buffer II. After lysis, the supernatant is collected by centrifugation at 10,000 g, 4 °C for 10 min. 20 µl of supernatant is diluted in ddH2O (1:5). Perform PCR reaction and heteroduplex digestion following the manufacturer's protocol. The diluted genomic DNA can be store at −20 °C for at least a year.

*Note: Conventionally, tail lysis buffer can also be used for sgRNA validation. However, the amplicon is preferably to be <800 bp, and a harsh condition tolerated DNA polymerase should be used. In our hands, Both KOD FX Neo (Toyobo #KFX-201) and Extaq (TaKaRa #RR001A) work well with tail lysis buffer. KOD FX Neo can also replace the DNA polymerase provided in the Guide-it Mutation Detection Kit, and the T7 endonuclease (NEB #M0302S) can be used to replace the Guide-it Resolvase.*

**Table:** Tail lysis (For 1–2 × 10^5^ cells)

Buffer I	
NaOH	25 mM
EDTA pH 8.0	2 mM
Buffer II	
Tris pH 5.5	40 mM

Add 50 µl Buffer I to cell pellet and incubate at 95 °C for 15 min

Neutralize the lysate with Buffer II (1:1 ratio), use directly for PCR

**Fig. 2:**
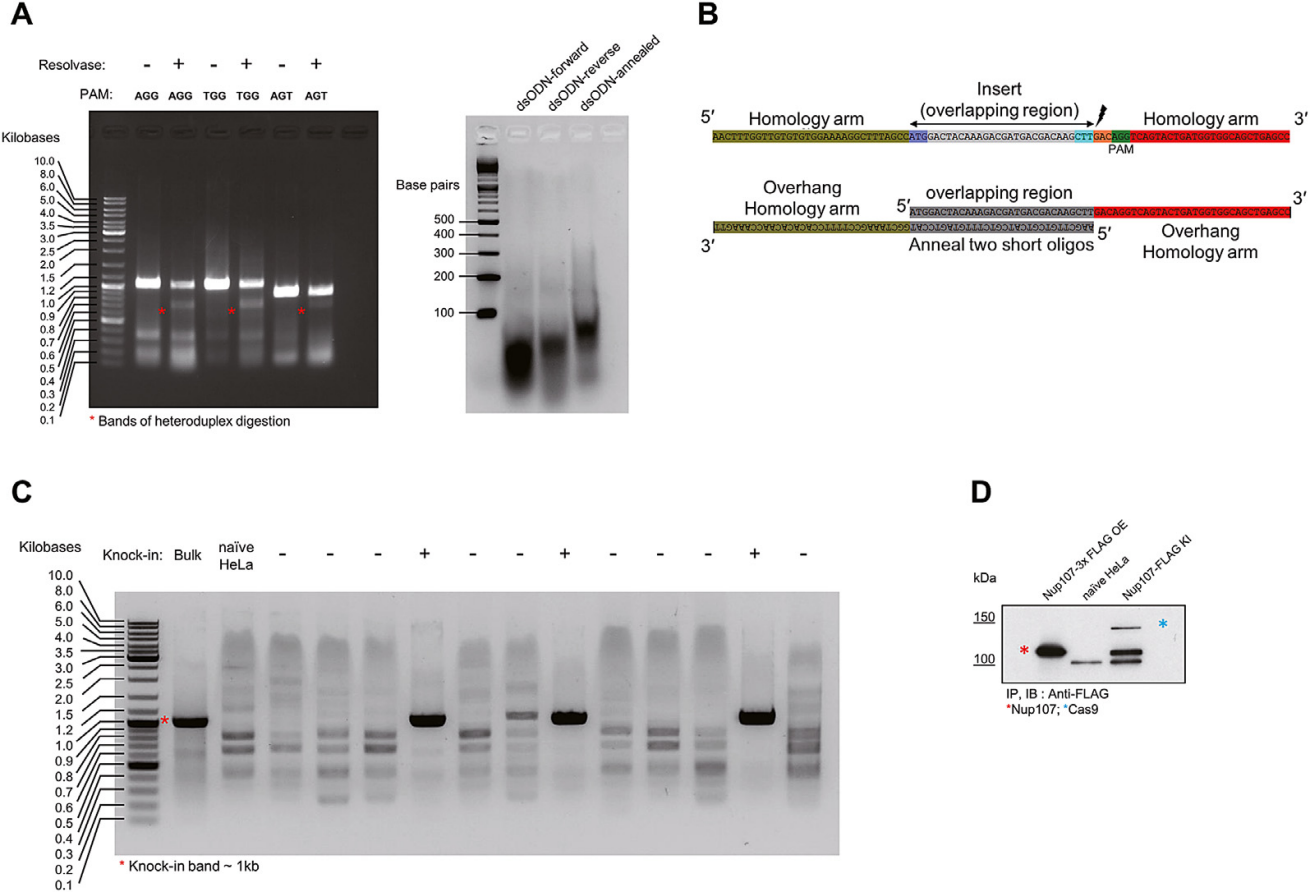
Workflow example and validation of the protocol A) Left panel: sgRNA validation by heteroduplex digestion. Electrophoresis of PCR products from genomic DNA extracts from different sgRNA containing Cas9 constructs. Heteroduplex digestion of these PCR products yielded an additional band. Right panel: Annealed semi-dsODN, a size-increased band could be observed. B) The design of semi-dsODN using AGG as a PAM sequence. The PAM homology arm was shown in red, and the non-PAM homology arm was shown in olive. This design contained a 30-nt overlapping region and a 32-nt homology arm. C) DNA electrophoresis of PCR products using a pair of insert-specific primers (expected size-1kb). Bulk served as a positive control and naive HeLa served as a negative control. This image contained three positive clones"+". D) Immunoprecipitation of Nup 107-FLAG protein using FLAG-M2 agarose gel (Sigma#A2220). The red asterisk was the exogenously expressed Nup107-3XFLAG protein. The blue asterisk was the FLAG-tagged Cas9 protein.

Run a gel electrophoresis to check the cleavage efficiency of sgRNA (see example in Fig. 2A). Generally, good sgRNA generates clear fragments after heteroduplex digestion.

Additional information: Since the epitope tag needs to be placed at either C-terminus or N-terminus of proteins, considering the insertion should be within +/− 10 bp of the double-strand break, choices of PAM sequence sometimes are very limited. In our example, we used the pL-CRISPR.EFS.tRFP, which requires the standard NGG as PAM sequence. If an NGG PAM cannot be found, one can consider using the pX330-SpCas9-NG (Addgene #117919) to avoid the PAM limitation. In both cases, sgRNA validation is recommended.

### II semi-dsODN design, transfection, and validation (5–6 days)

2.1 Based on the predicted DSB site of sgRNA, design semi-dsODN oligos (In our example of Nup107 tagging, we selected the AGG PAM shown in Fig. 2B).

*Caution: The design of the overhang region is critical for integration. We tagged the N-terminal of Nup107 with 1× FLAG sequence immediately after the ATG codon in the example provided. Overhang (homology arm) towards the PAM side means the same strand where the PAM sequence locates. In brief, homology arms are designed based on the PAM +3 position of the predicted Cas9 cutting site, extended 29 nt towards the PAM side – a total of 32 nt. For the complementary strand, the homology arm is 32 nt towards the non-pam side of the insert. The insert is designed as an overlapping region that forms a complementary double-stranded region (* Fig. 2 *B)*

*Note: In our hands, homology arms between 30 and 36 nt successfully integrated into the genome. Any shorter or longer arms have not been tested. Chemical modification at the 5*′ end *of semi-dsODN is not necessary for a successful knock-in.*

2.2 Order semi-dsODN from a commercial source (e.g., Eurofins Scientific [10 nmol scale in 50 µM concentration]). Anneal oligos using the same condition as the sgRNA oligos and adjust the final concentration to 0.5 µg/ul according to the DNA sequence (NEBioCalculator). Check the annealed semi-dsODN by electrophoresis (Fig. 2A).

2.3 Seed 0.5 × 10^6^ HeLa cells onto a 6 cm dish in DMEM medium one day before transfection. For each dish, transfect cells using the following

**Table:** 

sgRNA Cas9 construct	2 µg
semi-dsODN	0.5 µg
PEI	10 µl
Opti-MEM	100 µl
Mix and Incubate at room temperature for 5 min	

Add this mixture evenly onto the dish and replace medium after 6–8 h.

Secondary transfection (preferably 6–8 h after primary transfection):

**Table:** 

semi-dsODN	1 µg
PEI	4 µl
Opti-MEM	100 µl
Mix and Incubate at room temperature for 5 min	

Add this mixture evenly onto the dish and change medium the next day.

*Note: PEI is a toxic but cost-effective transfection reagent. To reduce the toxicity caused by a high PEI dose, delivery of semi-dsODN with two sequential transfections increases the transfection efficiency and overall cell survival.*

2.4 At 48 h after the secondary transfection, harvest cells (bulk) and divide into three tubes. One tube can be cryopreserved for future use (e.g., single-cell clones). One tube is used for crude genomic DNA extraction and PCR validation using an insert-specific primer (In our example is the FLAG sequence). Successful integration should yield a clear band (Fig. 2C). And one tube can be used for immunoblotting to confirm the tagged protein after PCR validation.

*Note: One can also validate the knock-in PCR product by restriction digestion if a specific (and unique) restriction site is inserted. In our example, a HindIII site was inserted after the FLAG tag so we could digest the PCR product with HindIII. However, it is better to perform this step after single-cell clone isolation as PCR reactions amplify mostly the non-inserted alleles if the bulk sample is used.*

2.5 Once the insert sequence is confirmed. Western blot can be performed using whole-cell lysate. Generally, 1x10^6^ cells are collected and lysed in 100 µl SDS lysis buffer. Boil the lysate at 95 °C for 5 min and dilute with 100 µl Tris diluent. Briefly sonicate the dilute sample with 15–20 pluses to reduce stickiness (depending on the machine output). After sonication, the sample is centrifuged at 10,000 g, 4 °C for 10 min. 80 µl of supernatant is mixed with 20 µl 5× Laemmli buffer and Boil at 95 °C for 5 min. The SDS-PAGE running information below is for protein Nup107 (107 kDa).

**Table:** 

SDS lysis buffer	

SDS	1% (w/v)
Tris pH 7.5	20 mM
NaCl	140 mM
Tris diluent	

Tris pH 6.8	20 mM
NaCl	20 mM
Glycerol	5% (v/v)

SDS PAGE (8%) – Tris-Glycine buffer with 0.1% SDS

Load 8 ul sample (lysate of 1 × 10^6^ cells), 120 V for 15 min, 160 V for 60 min

Wet transfer – Tris-Glycine buffer without SDS

Methanol activated PVDF membrane, 80 V 4 °C for 90 min

2.6 After transfer, block the membrane with 5% skimmed milk at room temperature for 30 min. For the FLAG tag, the blocked membrane is incubated with FLAG-M2 antibody (1:5000, Sigma #F3165) at 4 °C overnight with mild shaking. After incubation, wash the membrane with TBS-T (0.1% tween) for 15 min twice at room temperature. Incubate the membrane with secondary antibody (anti-mouse HRP, GE #NA931V) at room temperature for 1 h (or 4 °C overnight). After incubation of secondary antibody, wash the membrane with TBS-T for 15 min twice at room temperature and incubate the membrane with ECL solution (PerkinElmer #NEL104001EA) for 2 min. Remove excessive ECL solution and expose the membrane with X-ray film (or any applicable imaging system).

Additional information**:** For abundant proteins such as Nup107 and EIF4A3, enrichment by immunoprecipitation is not necessary. However, when protein expression level is unknown (or expression level is very low), the tagged protein might not be easily detected in the bulk cell lysate. In this case, a single-cell clone should be established first (see Optional Step III), or immunoprecipitation should be performed (Fig. 2D).

#### III Optional

**Single-cell clone establishment (*can be added between*** steps 2.3***–2.4)***

**3.1** At 24 h after secondary transfection, RFP positive cells can be sorted by flow-cytometry. 10,000 cells from the top 20% RFP positive population are collected (if pL-CRISPR.EFS.tRFP is used). Generally, 300 cells are plated on a 6 cm dish for colony picking (one 6 cm dish for 16–24 colonies). The rest of the sorted cells can be expanded for few days to check by PCR and Western blot to confirm the knock-in before colony picking (follow steps 2.4–2.6).

*Note: It is recommended to check the RFP positive population (sorted bulk) before proceeding to more labor-intensive steps. Also, instead of using flow-cytometry to enrich the transfected population, antibiotic selection can also be performed using other CRISPR-Cas9 vectors (i.e., pL-CRISPR.EFS.PAC Addgene #57828).*

**3.2** After visible colonies are identified, prepare a 96-well dish (round-bottom, for dissociation of colonies) with 50 µl trypsin added on each well and a 24-well dish (for dissociated colonies) with 1 ml medium per well. Before picking colonies, wash the 6 cm dish with PBS once and then add 5 ml PBS to cover the whole dish. Set a 20 µl pipette to 10 µl and pick colonies by gentle scraping with suction. Transfer the colony into the 96-well dish. Try not to exceed 5 min for one 6 cm dish while picking. Incubate the 96-well dish at 37 °C for 5 min to dissociate the colonies. Neutralize trypsin with 100 µl medium containing 20% FBS and transfer dissociated cells to the pre-warmed 24-well dish prepared previously. Return the 24-well dish to the incubator after transfer and allow cells to expand for few days.

**3.3** Once cells reach 70–80% confluence. Cells can be harvested for PCR screening. Prepare a 24-well dish (dish A) with 1 ml medium per well and pre-warm at 37 °C. Label and wash the 24-well dish (dish B) containing cells with PBS once and dissociate cells with 150 µl trypsin for 5 min at 37 °C. Neutralize trypsin with 1 ml medium and transfer 1 ml dissociated cells (150 µl left in the well) to an Eppendorf tube for genomic DNA extraction (step 1.8). Draw 1 ml medium from the pre-warmed dish A to collect all the remaining cells from dish B. Be sure to label all tubes and wells in the same manner.

*Note: Not all colonies grow at the same pace. For convenience, harvest wells that are 70–80% confluent, extract genomic DNA and store them at −20* *°C before PCR screening.*

**3.4** Once specific PCR product of single-cell colony is confirmed (Fig. 2C). Western blot can be performed as steps 2.5–2.6.

*Note: Normally, it takes another 3–4 days to get enough cells for Western blot.*

## Single-cell clone sequencing

**Fig. 3:**
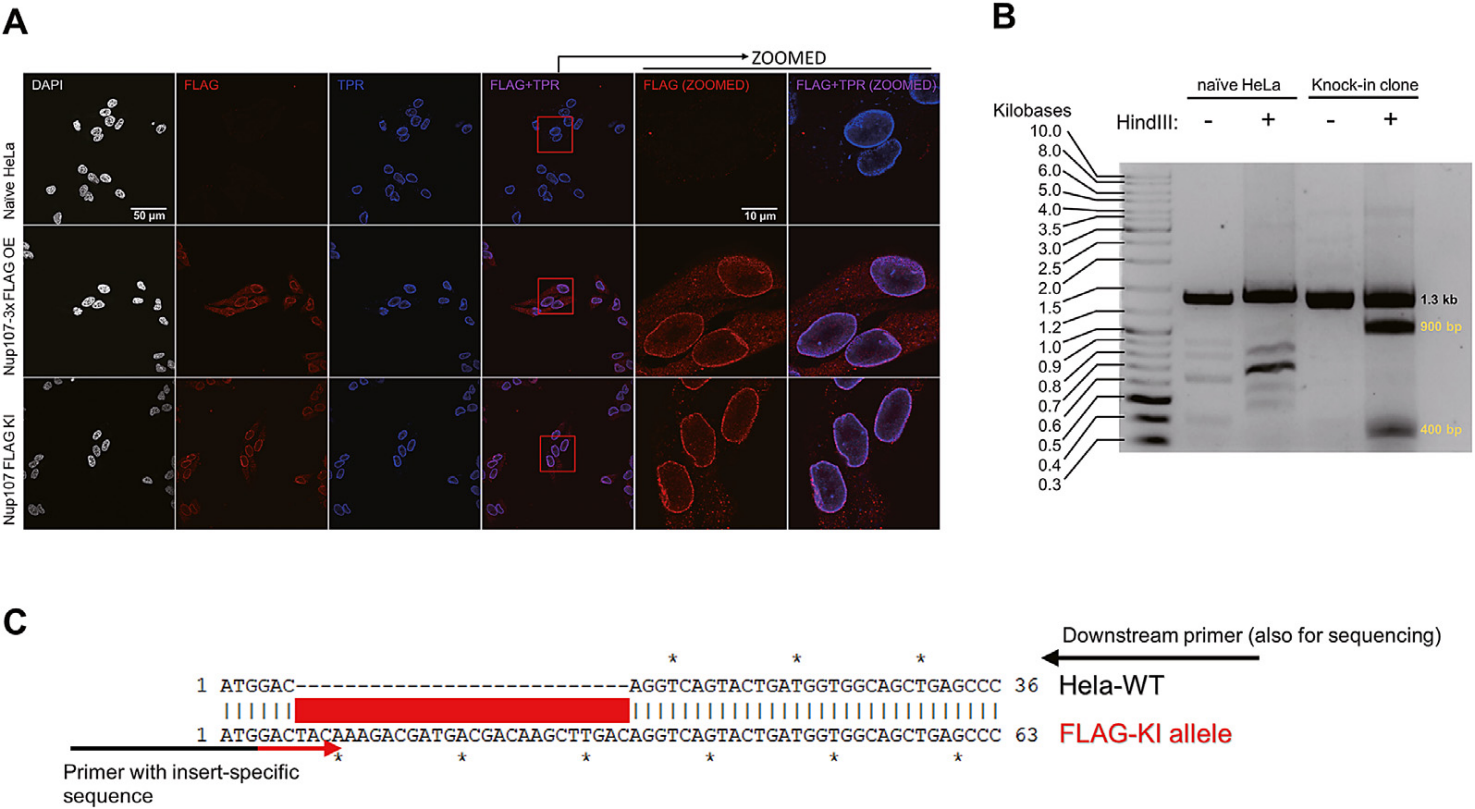
Validation of knock-in FLAG epitope in Nup107. A) Subcellular localization of Flag-tagged Nup107. Exogenously expressed Nup107-3xFLAG was introduced by retrovirus infection. The nuclear membrane was stained with TPR(BETHYL#A300-828A, shown in blue); Nup107-FLAG/3xFLAG was detected by FLAG-M2 antibody (Sigma#F3165, shown in red). Alexa Flour 488 and 568 (Thermo Fisher Sceintific A-32723 and A-11011) were used as fluorophores. Last two columns were zoomed images of the red square portion in the third right panels. B) Validation of the HindIII site insertion by electrophoresis. In knock-in cells, the 1.3 kb PCR product can be digested by HindIII, resulted in one 900 bp and one 400 bp fragment. C) Example PCR scheme for a sequencing based validation.

A commercially available TOPO TA cloning kit (Thermo Fisher Scientific) with Sanger sequencing can be used to obtain the sequence-based genetic information of knock-in clones. Also, a cost-effective alternative is to perform PCR reaction with an insert-specific primer followed by Sanger sequencing. In our example, an insert-specific primer was paired with a downstream primer to amplify the specific knock-in sequence (Fig. 3C). Sanger sequencing used the downstream primer to obtain detailed genetic information of the entire PCR product (usually a few nucleotides upstream of ATG).

## Method validation

Validation of the above protocol was demonstrated on a successful isolated knock-in clone. Compared to the exogenously overexpressed Nup107-3x FLAG, endogenous knock-in cells provided a cleaner background with appropriate subcellular localization maintained (Fig. 3A). Overall, from the 38 colonies we screened, 11 showed PCR positive (~28%), and 4 showed correct subcellular localization (nuclear membrane). In addition, restriction enzyme digestion using the inserted HindIII site and sequence-based validation were also performed (Fig. 3B and C). We also applied this method on proteins EIF4A3 and mtRF1L using HEK239T cells. We could detect the expected insertions by PCR reaction using the bulk DNA extracts (sgRNA sequences are HDR template sequences available upon request).

## Additional information

### Unpredictable Integration

*This method does not aim to improve the precision of gene knock-in. Edited cells generated by this method also suffer from genetic diversity. We have also detected unwanted mutations by Sanger sequencing.*

### Stem cell knock-in efficiency

*In our hands, stem cells are not well adapted for this method. The success rate of using this protocol is very low, presumably due to the low transfection efficiency. For this matter, following the original protocol for stem cell knock-in is recommended.*

*For primers used in this protocol, please see the supplementary table X1.*

## Declaration of Competing Interest

The authors declare no known competing financial interests or personal relationships that could have appeared to influence the work reported in this paper.

## Appendix Supplementary materials

Image, application 1

Image, application 2

## References

[1] Liang X., Potter J., Kumar S., Ravinder N., Chesnut J.D. (2017). Enhanced CRISPR/Cas9-mediated precise genome editing by improved design and delivery of gRNA, Cas9 nuclease, and donor DNA. J. Biotechnol..

[2] Richardson C.D., Ray G.J., DeWitt M.A., Curie G.L., Corn J.E. (2016). Enhancing homology-directed genome editing by catalytically active and inactive CRISPR-Cas9 using asymmetric donor DNA. Nat. Biotechnol..

[3] Ran F.A., Hsu P.D., Wright J., Agarwala V., Scott D.A., Zhang F. (2013). Genome engineering using the CRISPR-Cas9 system. Nat. Protoc..

