# Supplementary material for: A cost efficient protocol to introduce epitope tags by CRISPR-Cas9 mediated gene knock-in with asymmetric semi-double stranded template
Source: MethodsX. 2021 Apr 27;8:101365. doi: 10.1016/j.mex.2021.101365 (PMC8374323; doi:10.1016/j.mex.2021.101365)
Supplement: Supplementary file 1 [file mmc1.pdf]

Supp review table 1.
Price for making 1x FLAG-tag Knock-in template oligonucleotide:

Vender name	ssODN* 204 nt	ssODN** 151 nt	Semi-dsODN*** 54 nt	ssODN**/ Semi-dsODN***
$Sigma	N/A	18,120 JPY	1,064x2 JPY	x8.5
			2,128 JPY	
#IDT	65000 JPY	36,240 JPY	728x2 JPY	x24.9
			1,456 JPY	
$Eurofin	N/A	9,860 JPY	560x2 JPY	x8.8
			1,120 JPY	
#Price from their Japanese website				
$Sigma and Eurofin are special contract price				
1 JPY = 0.0092 USD (Mar. 18, 2021)				
